# Eating behaviors among chinese older adults: A qualitative study using the capability, opportunity, motivation, and behavior model

**DOI:** 10.1016/j.pmedr.2025.103156

**Published:** 2025-06-29

**Authors:** Qian Wang, Qian Li

**Affiliations:** Nursing Department, Affiliated People's Hospital of Jiangsu University, Zhenjiang City, Jiangsu Province, China

**Keywords:** Aged, Emotional eating, Qualitative research, Eating behavior, COM-B model, Digital divide, China

## Abstract

**Objective:**

This study explored the mechanisms underlying eating behaviors among Chinese older adults using the COM-B model, which conceptualizes behavior through Capability, Opportunity, and Motivation components.

**Methods:**

A qualitative descriptive design was adopted. Semi-structured interviews were conducted with 20 community-dwelling older adults in Zhenjiang, Jiangsu Province, China, from October to December 2024. Thematic analysis was employed to analyze the data.

**Results:**

Four major themes were identified: Restrictive Eating, Emotional Eating, Nutritional Literacy, and External Eating. Dietary patterns were influenced by physical health conditions, emotional states, family dynamics, and digital barriers to food information access.

**Conclusions:**

Eating behaviors among older adults are shaped by complex and interconnected factors across individual, social, and environmental domains. Interventions promoting healthy aging should integrate strategies to enhance capability, optimize opportunity, and strengthen motivation.

## Highlights

• Behavioral mechanisms of older adults' eating shaped by capability, opportunity, motivation.
• Nutritional Literacy strongly influence dietary behaviors.
• Limited digital skills reduce access to healthy food options.
• Family dynamics both support and limit dietary autonomy.
• Findings guide culturally tailored nutrition interventions for older adults.

## 1 Introduction

The rapid aging of China's population presents significant socio-economic challenges. By 2022, 267 million people (18.9 %) were aged 60 or above, projected to exceed 30 % by 2035 (United Nations, Department of Economic and Social Affairs, Population Division, 2022). The health status of the elderly is a critical factor influencing sustainable development and family well-being (Lee et al., 2024). Currently, older adults face a “double burden of malnutrition”—undernutrition leading to sarcopenia and osteoporosis (Vetta et al., 1999), and overweight/obesity increasing risks of chronic diseases (Keramat et al., 2021). Eating behavior is a pivotal determinant of nutritional status and overall health.

Eating behavior encompasses the actions and psychological processes involved in food acquisition, selection, preparation, and consumption (Sugiharto et al., 2021). In the elderly, these behaviors are shaped by a complex interplay of physiological (e.g., tooth loss, diminished taste), psychological (e.g., loneliness), social, and cultural factors (Antoniadou and Varzakas, 2021; Walker-Clarke et al., 2022; Mercille et al., 2012).

Existing tools, such as the Dutch Eating Behavior Questionnaire (DEBQ) and the Three-Factor Eating Questionnaire (TFEQ), assess dimensions like restrictive eating, emotional eating, and external eating (Van Strien et al., 1986; Karlsson et al., 2000), but often overlook broader influences (e.g., food choice, preparation, and behavioral mechanisms), especially those arising from aging-related changes.

Although the Dutch Eating Behavior Questionnaire was originally designed to explore obesity-related behaviors, its foundations—Restraint Theory, Emotion Regulation Theory, and Externality Theory—reflect universal behavioral mechanisms applicable across populations (Van Strien et al., 1986). Recent review (Walker-Clarke et al., 2022; Caso and Vecchio, 2022) confirm the relevance of these constructs to older adults: dietary restraint linked to chronic disease management, emotional eating driven by life transitions, and external eating shaped by social and cultural contexts—independent of obesity status. However, no unified theoretical framework has been systematically applied to integrate these insights for older adults.

In China, traditional values (e.g., frugality) and social structures significantly influence eating behaviors (Huang et al., 2021). Frugality may drive excessive restriction, while family gatherings promote overeating (Wu et al., 2023). Economic limitations also affect access to diverse, nutritious foods (Wu et al., 2023). Yet few studies have systematically explored how socio-cultural, environmental, and individual factors interact to shape elderly eating behaviors.

To address this gap, this study adopts the COM-B model (Capability, Opportunity, Motivation - Behavior) (Armitage et al., 2024) as its guiding framework. COM-B conceptualizes behavior as resulting from interactions between capability, referring to an individual's physical and psychological capacity to perform the behavior; opportunity, encompassing external factors that facilitate or hinder the behavior; and motivation, involving internal processes that energize and direct behavior, including both reflective and automatic mechanisms.

Widely used across public health domains, COM-B provides a flexible framework for analyzing complex behaviors. In this study, it offers a structured lens to examine how capability (e.g., nutritional knowledge, emotional coping), opportunity (e.g., family dynamics, digital access), and motivation (e.g., health goals, cultural identity) shape older adults' eating behaviors. The Dutch Eating Behavior Questionnaire domains informed interview guide development, while the analysis remained open to emergent themes.

The objective is to explore behavioral mechanisms underlying eating behaviors among Chinese older adults through the COM-B model, with emphasis on the dynamic interplay of capability, opportunity, and motivation. Findings aim to inform effective, culturally sensitive dietary interventions for aging populations.

## 2 Research methods

### 2.1 Theoretical framework and analytical approach

This study was guided by the COM-B model, which conceptualizes behavior as the dynamic interaction of Capability, Opportunity, and Motivation. The model informed both the development of the semi-structured interview guide and the analytical framework for exploring elderly eating behaviors.

In addition, theoretical constructs from the Dutch Eating Behavior Questionnaire—Restraint Theory, Emotion Regulation Theory, and Externality Theory—served as an initial heuristic to inform specific interview questions. This approach drew upon established constructs relevant to dietary behavior while remaining open to emergent themes beyond the Dutch Eating Behavior Questionnaire domains. Notably, participants were community-dwelling older adults with varied health and weight statuses, not limited to individuals with obesity.

Aligned with a constructivist epistemological stance, data collection and analysis remained open to themes beyond predefined COM-B categories. An inductive thematic analysis approach allowed participants' lived experiences and socio-cultural contexts to shape the thematic structure. The COM-B model subsequently served as a sensitizing and interpretive framework to deepen understanding of the findings.

### 2.2 Semi-structured interview design

The interview guide was developed iteratively through theoretical grounding, literature review, and expert consultation. The panel included three senior nutrition and public health researchers, two gerontology experts, and two qualitative methodologists, ensuring content relevance, cultural appropriateness, and alignment with the COM-B framework.

Pilot interviews with two participants assessed feasibility, clarity, and relevance. Feedback informed refinements to wording, sequencing, and content to enhance comprehensibility.

The finalized interview guide targeted three primary behavioral domains—restrictive eating, emotional eating, and external eating—each mapped onto the Capability, Opportunity, and Motivation components of the COM-B model **(**Table 1**).** This design allowed for both theory-informed inquiry and flexibility to capture emergent themes.

**Table 1:** Semi-Structured Interview Guide for Exploring Eating Behaviors Among Older Adults in Zhenjiang, China (2024), Mapped to Capability, Opportunity, Motivation–Behavior Model Components.

Behavioral Domain	COM-B Component	Sample Questions
Restrictive Eating	Capability	- How do your health conditions (e.g., oral health, chronic diseases) affect your food choices? - Do you feel you have enough nutritional knowledge to guide your dietary choices?
	Opportunity	- Do your family members impose any restrictions or give advice about your diet? - Are there any community resources (e.g., health programs) that help you manage your diet?
	Motivation	- What motivates you to consciously control your food intake? - How do you perceive the importance of restricting certain foods in your daily life?
Emotional Eating	Capability	- When you feel stressed, lonely, or sad, do you have alternative coping strategies besides eating? - How confident do you feel in managing your emotions without relying on food?
	Opportunity	- Do social events or gatherings influence your emotional eating? - Are there situations where your social environment encourages you to eat emotionally?
	Motivation	- How do emotional changes (such as happiness, sadness, or stress) drive your eating behavior? - Can you describe situations where eating helped you feel better emotionally?
External Eating	Capability	- How much control do you feel you have over external influences like the sight or smell of food? - Are you aware when external stimuli influence your food choices?
	Opportunity	- How accessible are healthy food options in your living environment (e.g., markets, restaurants)? - Does the local food environment make it easier or harder for you to eat healthily?
	Motivation	- Have you ever been influenced by food advertisements, promotions, or others' suggestions to eat something you hadn't planned? - In what situations are you more likely to change your food choices because of external factors?

## 3 Study procedures

### 3.1 Study context and data collection

Data collection occurred in Zhenjiang, Jiangsu Province, China. The city's mix of urban and rural settings and sizable elderly population provided a rich socio-cultural context.

Interviews took place at participants' homes or community centers, based on preference, fostering a comfortable environment for open discussion. All interviews were audio-recorded with informed consent and supplemented by field notes on non-verbal cues and context. Before the interview, participants completed a brief questionnaire on demographics, including age, sex, education, living arrangement, and health status.

### 3.2 Participant recruitment and sampling strategy

Purposive sampling was used to maximize diversity in sex, age, socio-economic status, living arrangements, and health conditions (presence/absence of chronic diseases). Inclusion criteria were: (1) age ≥ 60 years; (2) residence in Zhenjiang for ≥5 years; (3) ability to articulate personal experiences.

Participant recruitment took place between October 23, 2024, and December 9, 2024. Community health workers and local organizations facilitated recruitment through flyers, referrals, and direct invitations during health education sessions and senior activity groups. Recruitment sites included community health service centers, elderly day-care facilities, and public health lectures. Snowball sampling further enhanced sample diversity via community networks.

Twenty participants were interviewed, with sample size guided by thematic saturation. Seventeen interviews were face-to-face and three via video call, ranging from 20 to 45 min (average ≈ 35 min). In two cases, participants requested family/friend support; one eligible participant declined. The diverse sample enhanced the transferability of findings.

### 3.3 Statistical analysis

Thematic analysis followed Braun and Clarke's six-phase approach (Braun and Clarke, 2023). A combined inductive-deductive coding strategy was used. Inductively, patterns and themes were allowed to emerge naturally, consistent with the study's constructivist stance. The COM-B model concurrently served as a deductive sensitizing framework, guiding the interpretation of behavioral mechanisms. Two researchers independently conducted open coding, resolving discrepancies through consensus. A coding log and memos ensured transparency.

Codes were grouped into candidate themes, reviewed, and refined into a coherent structure. Representative quotes were selected to illustrate key findings.

All interview transcripts were transcribed verbatim in simplified Chinese within 48 h after data collection and imported into NVivo 12 Pro software for coding and analysis.

## 4 Results

### 4.1 Participants' characteristics

Table 2 summarizes the characteristics of the 20 participants (12 females, 8 males), aged 60–94 years. Participants varied in living arrangements (with spouse, with children, alone), economic status (low to middle income), education (illiterate to university), occupations (farmer, worker, civil servant), and health conditions (hypertension, diabetes, Chronic Obstructive Pulmonary Disease, cancer, or good health). This diversity supports the transferability of findings.

**Table 2:** Demographic and Health Characteristics of Older Adult Participants (*N* = 20), Zhenjiang, China, October–December 2024.

ID	Age	Gender	Living Situation	Economic Status	Education Level	Health Condition	Occupational History
1	66	Female	Lives with spouse	Middle income	High school	Osteoporosis	Worker
2	75	Male	Lives with spouse and child	Middle income	University	Hypertension	Civil servant
3	72	Female	Lives alone	Low income	Primary school	Post-surgery (stomach cancer)	Farmer
4	73	Female	Lives with spouse and child	Middle income	High school	Diabetes	Civil servant
5	67	Male	Lives with spouse	Middle income	High school	Good health	Teacher
6	70	Female	Lives with spouse and child	Low income	Primary school	Diabetes, Hypertension	Worker
7	67	Female	Lives with spouse	Middle income	Illiterate	Hypertension	Caregiver
8	70	Female	Lives with spouse	Middle income	Illiterate	Good health	Caregiver
9	68	Male	Lives with spouse	Middle income	Middle school	Diabetes	Worker
10	77	Female	Lives alone	Middle income	Middle school	Chronic Obstructive Pulmonary Disease (COPD), Hypertension	Worker
11	63	Female	Lives with spouse	Low income	Primary school	Osteoporosis	Farmer
12	64	Male	Lives with spouse	Middle income	Middle school	Good health	Businessman
13	62	Female	Lives with spouse and child	Low income	Illiterate	Good health	Caregiver
14	60	Female	Lives with spouse and child	Low income	High school	High blood sugar	Worker
15	81	Female	Lives with spouse	Low income	Illiterate	Hypertension, Depression, Gallstones	Worker
16	87	Female	Lives with spouse and child	Middle income	Primary school	Good health	Worker
17	94	Male	Lives alone	Middle income	University	COPD, Lung Cancer, Stroke, Osteoporosis, Prostate Hyperplasia	Factory secretary
18	62	Female	Lives with spouse	Low income	Primary school	Good health	Sanitation worker
19	75	Male	Lives with spouse	Low income	Illiterate	Pulmonary infection, Hypertension, Hypokalemia	Farmer
20	71	Female	Lives with child	Middle income	Primary school	Good health	Farmer

### 4.2 Thematic findings

**Fig. 1:**
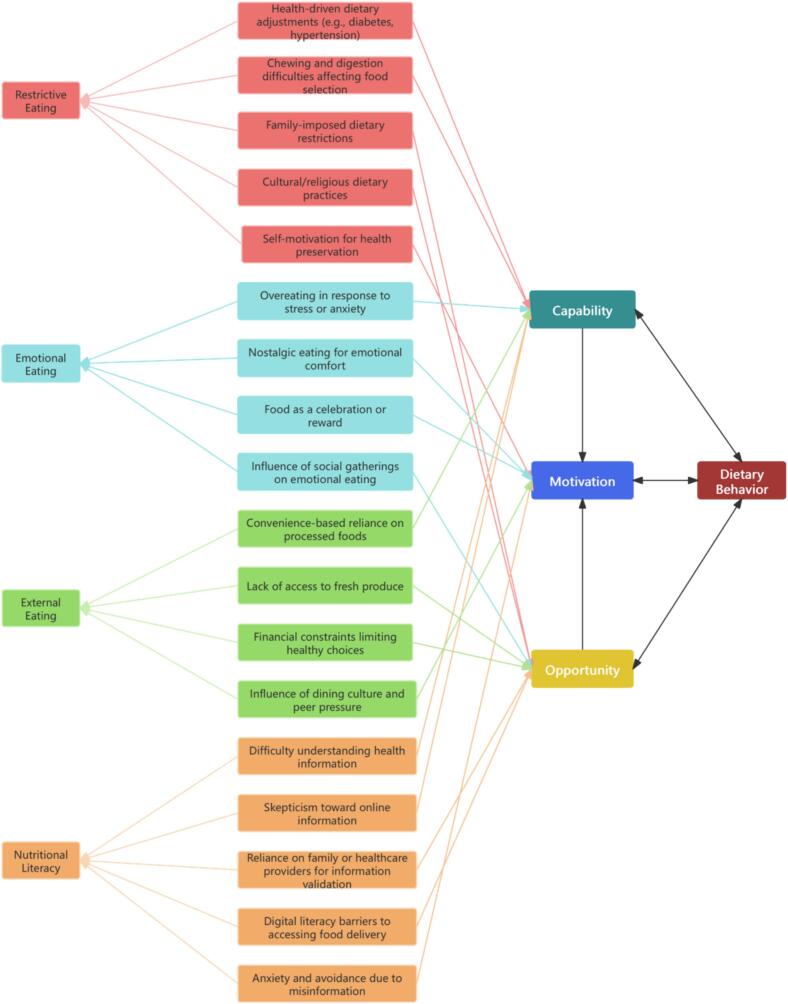
Thematic Mapping of Eating Behaviors Among Older Adults to Capability, Opportunity, Motivation–Behavior Model Components, Zhenjiang, China (2024).

Notably, “Literacy Barriers” emerged inductively, as participants raised this theme during discussions of dietary behaviors. Thematic analysis identified four domains of eating behaviors: Restrictive Eating, Emotional Eating, Nutritional Literacy, and External Eating, each mapped to the Capability, Opportunity, and Motivation components of the COM-B model. Fig. 1 illustrates the dynamic interactions among these factors. Detailed subthemes and mappings are provided in **Supplementary Table 1**.

#### 4.2.1 Restrictive eating

Restrictive eating behaviors among elderly participants were shaped by the dynamic interplay of Capability (individual health conditions), Opportunity (social influences), and Motivation (personal health goals).

#### 4.2.2 Capability factors

Health conditions such as diabetes, hypertension, and hyperlipidemia limited participants' ability to consume certain foods, necessitating self-regulation in their dietary practices.

“Since being diagnosed with diabetes, I've stopped eating sweets altogether. I can't even enjoy a small piece of candy without worrying about my sugar levels.” (P1, Male, 65).

“My cholesterol levels have been high for years, so I've completely cut out fried foods.” (P3, Female, 72).

Dental problems and chewing difficulties further constrained food choices, often reducing dietary diversity.

“Because of my teeth, I can't eat anything too hard anymore, even if I want to.” (P7, Female, 71).

Participants emphasized the frustration of being physically unable to consume traditional or preferred foods, highlighting how physiological decline affected dietary autonomy.

#### 4.2.3 Opportunity factors

Family members exerted substantial control over dietary choices (Opportunity), which in turn interacted with participants' personal motivations to maintain or improve health.

“My son controls the salt and oil at home; he says it's healthier for me.” (P2, Female, 70).

While family-imposed restrictions provided external support, they also sometimes conflicted with participants' preferences or cultural food practices.

“We always avoid meat during certain festivals; it's part of our tradition.” (P12, Female, 67).

This dynamic between external control and personal motivation shaped participants' adherence to dietary restrictions.

#### 4.2.4 Motivation factors

Participants demonstrated self-initiated motivation to proactively restrict their diets for perceived health benefits, which often reinforced or complemented family-imposed dietary practices (Opportunity). In some cases, Motivation drove more stringent restrictions than those recommended by family or physicians.

“I want to live longer for my grandchildren, so I try to eat less fried food.” (P6, Male, 74).

These Motivation-Capability-Opportunity interactions contributed to sustained dietary behavior change.

#### 4.2.5 Emotional eating

Emotional eating behaviors among older adults reflected a dynamic interaction between emotional coping capacities (Capability), situational triggers and social contexts (Opportunity), and internal emotional drivers (Motivation).

#### 4.2.6 Capability factors

Participants with limited emotional coping skills often turned to food for immediate stress relief, demonstrating how deficits in emotional regulation capacity contributed to maladaptive eating patterns.

“When I'm upset, I find myself eating whatever snacks are around.” (P5, Female, 73).

#### 4.2.7 Opportunity factors

Social gatherings provided situational opportunities for emotional overeating, where festive atmospheres and social norms encouraged increased food intake. These external cues interacted with individual coping capacities and motivations.

“During family gatherings, I always end up eating more than I should.” (P11, Male, 69).

#### 4.2.8 Motivation factors

Food served as a powerful emotional regulator, offering comfort, nostalgia, and reward. Such motivational drivers reinforced the tendency to engage in emotional eating when opportunities arose and when coping capacities were limited.

“Making dumplings reminds me of happier times with my family.” (P10, Female, 70).

“After a hard day, I reward myself with my favorite cake.” (P19, Male, 65).

Thus, Motivation-Capability-Opportunity interactions were evident in shaping emotional eating behaviors among participants.

#### 4.2.9 External eating

External eating behaviors were shaped by the physical and social environments in which older adults lived, in interaction with their capabilities and motivational orientations**.**

#### 4.2.10 Capability factors

Physical limitations such as fatigue, mobility issues, and the burden of meal preparation contributed to increased reliance on convenient, often less healthy, food options**.** These limitations interacted with environmental opportunities to shape dietary behaviors.

“Cooking for just one person is too much trouble, so I buy frozen meals.” (P8, Female, 75).

#### 4.2.11 Opportunity factors

Environmental and economic constraints—including limited access to fresh produce and high costs of healthy foods—further restricted healthy eating choices.

“There aren't many fresh vegetables at the market near my home.” (P9, Male, 70).

“Organic food is so expensive. I usually just buy whatever is cheaper.” (P13, Female, 66).

Social dining environments also presented opportunities for external eating behaviors influenced by cultural traditions and peer norms.

“We meet at the same restaurant every week. It's not about the food; it's about the company.” (P10, Female, 68).

#### 4.2.12 Motivation factors

Motivational factors such as the desire to fit in socially and participate fully in group activities influenced external eating choices, often overriding personal dietary intentions or health considerations.

“When everyone else orders rich dishes, I don't want to be the odd one out.” (P4, Male, 68).

These patterns illustrate how Opportunity, Capability, and Motivation components dynamically interact to shape external eating behaviors in elderly populations.

#### 4.2.13 Nutritional literacy

Nutritional Literacy emerged as an important cross-cutting factor influencing elderly dietary behaviors, encompassing functional, interactive, and critical dimensions. It primarily mapped to Capability (knowledge and skills), but also interacted with Opportunity (access to reliable information and digital platforms) and Motivation (confidence, fear of misinformation).

#### 4.2.14 Capability factors

Participants often expressed difficulties in understanding scientific nutritional information, finding terms and concepts overwhelming.

“All these talks about calories and cholesterol are too complicated for me.” (P5, Female, 72).

Some participants demonstrated skepticism toward online health information due to past experiences with misleading advertisements.

“There are too many health advertisements. I don't believe half of them.” (P9, Female, 67).

These limitations constrained their ability to make informed food choices independently.

#### 4.2.15 Opportunity factors

Family members and healthcare providers were frequently relied upon as trusted sources to validate dietary information.

“I always ask my daughter before trying anything new I read online.” (P20, Male, 69).

Importantly, although online food delivery platforms and health information resources have become increasingly available in China, many elderly participants faced significant digital literacy barriers. They reported difficulty using smartphones and navigating delivery applications, limiting their ability to access a broader range of convenient, potentially healthier food options.

“I see all those apps, but I don't know how to order. I still have to go to the market myself.” (P16, Male, 72).

Thus, a digital divide exacerbated inequalities in nutritional opportunity: despite the theoretical availability of services, limited digital skills effectively restricted access for many elderly individuals. These barriers underscored how external resources interact with individual Capability to shape dietary decision-making.

#### 4.2.16 Motivation factors

Fear of making dietary mistakes and anxiety over conflicting information led to avoidance behaviors. Such motivational states often exacerbated Capability limitations, reducing dietary adequacy.

“I'm so afraid of eating the wrong thing that I sometimes just eat very little.” (P14, Female, 68).

Thus, Motivation and Capability factors interacted to influence not only information processing but also actual dietary practices.

## 5 Discussion

This study applied the COM-B model to explore how Capability, Opportunity, and Motivation interact to shape restrictive eating, emotional eating, external eating, and Nutritional Literacy among older adults. While the Dutch Eating Behavior Questionnaire domains informed the interview guide, the analysis was grounded in COM—B, highlighting that eating behaviors emerge from dynamic interactions within sociocultural contexts.

### 5.1 Restrictive eating: Capability and opportunity constraints

Health-related limitations (e.g., chronic diseases, chewing difficulties) were key drivers of restrictive eating, necessitating dietary adjustments that reduced food diversity and enjoyment (Kiesswetter et al., 2018). External factors, such as family-imposed restrictions and cultural practices, further shaped these behaviors. While often well-intentioned, family control sometimes conflicted with personal autonomy, echoing intergenerational caregiving tensions (Subramaniam and Mehta, 2024). Interventions should balance health guidance with respect for autonomy and cultural identity, as Opportunity constraints can both complement and conflict with individual Motivation and Capability.

### 5.2 Emotional eating: Emotional coping and social opportunity

Emotional eating was driven by limited emotional coping (Capability) and social situational triggers (Opportunity). Participants used food to cope with stress, loneliness, and loss, highlighting its role as an emotional regulator. Positive social occasions, such as family gatherings, further reinforced this behavior through shared cultural rituals (Fuente González et al., 2022; Stockbridge et al., 2023). Interventions should address emotional vulnerabilities while promoting healthier communal eating practices, as emotional coping, social cues, and internal Motivation interact to sustain emotional eating.

### 5.3 External eating: Environmental, economic, and digital access constraints

External eating behaviors were shaped by Opportunity-related factors, including limited access to fresh produce, financial barriers, and reliance on convenience foods—often contributing to suboptimal dietary patterns (Davies and Reid, 2024). Participants living in poor food environments reported constrained healthy choices, compounded by financial limitations.

Although food delivery services and digital grocery platforms have expanded in China, many elderly participants faced digital literacy barriers (Xu et al., 2024), excluding them from these resources. This highlights a modern Opportunity inequality, where digital exclusion further restricts access to healthy food, even when physically available.

Social dining contexts also introduced peer pressure to conform to unhealthy eating norms, especially during communal meals.

To promote healthier external eating, interventions must address structural food inequalities, reduce digital barriers, and foster social environments that balance tradition with health consciousness. The interplay of Opportunity limitations (physical and digital), Capability constraints, and social Motivations (peer norms) reinforces external eating patterns, often misaligned with health goals.

### 5.4 Nutritional literacy: A critical capability and motivation mediator

A key contribution of this study is the identification of Nutritional Literacy as a cross-cutting behavioral determinant. While primarily linked to Capability (knowledge and skills), it also interacts with Opportunity (access to reliable information, digital literacy, social supports) and Motivation (fear of misinformation, desire for autonomy).

Participants reported difficulties understanding complex nutrition information, navigating contradictory media, and judging the credibility of online content. These challenges, combined with motivational factors—such as fear of misinformation—led to food avoidance, overly restrictive diets, and anxiety about “eating incorrectly.”

The growing complexity of the digital information environment further exacerbates these challenges for older adults, whose dietary behaviors increasingly depend on their ability to critically access, evaluate, and apply nutritional knowledge (Luo et al., 2024). In particular, interactive and critical dimensions of Nutritional Literacy (evaluating online information and navigating social influences) are shaped by both Opportunity and Motivation, highlighting the dynamic interplay of COM-B components in shaping information-driven eating behaviors.

Enhancing Nutritional Literacy—especially critical appraisal and digital navigation skills—could empower older adults to make informed, autonomous dietary choices, bridging the gap between information abundance and effective health action.

### 5.5 Implications for practice and policy

The COM-B model offers a comprehensive framework for understanding not only what dietary behaviors occur among older adults, but also why and how they are sustained or modified within real-world sociocultural contexts—advancing beyond traditional tools such as the Dutch Eating Behavior Questionnaire or the Three-Factor Eating Questionnaire, which focus mainly on behavioral outcomes. This holistic perspective is particularly valuable in complex domains like elderly eating behaviors, where structural, social, and emotional factors intersect.

Grounded in this COM-B framework, the present findings suggest that promoting healthy eating among older adults requires multifaceted interventions that simultaneously address the three core components—Capability, Opportunity, and Motivation.

Capability enhancement should prioritize the development of accessible educational initiatives to improve health literacy, strengthen emotional coping skills, and teach adaptive cooking techniques suited to physiological limitations (e.g., preparing softer foods). Additionally, targeted digital literacy training should be incorporated to empower older adults to navigate modern food systems and critically evaluate online nutritional information.

Opportunity optimization calls for policy reforms that foster age-friendly food environments, improving the affordability and accessibility of nutritious foods not only in physical spaces but also through digital platforms. Furthermore, community-based initiatives should cultivate supportive social contexts that encourage healthy communal eating while minimizing negative peer pressure.

Motivation strengthening should focus on designing behavior change strategies aligned with older adults' intrinsic values—such as health preservation, familial bonding, and cultural continuity—while helping them manage anxiety arising from conflicting or overwhelming health information.

Together, these integrated and culturally sensitive approaches are critical to supporting sustainable dietary behavior change and promoting healthy aging among older populations in the context of evolving social and technological landscapes.

### 5.6 Future research directions

Future research should prioritize quantitative studies to validate and extend these qualitative findings, clarifying the relative contributions of Capability, Opportunity, and Motivation factors across diverse settings. Longitudinal designs are needed to explore how these interactions evolve over time in relation to life transitions, digital adoption, and changing food environments.

Additionally, emerging technologies—such as AI-driven personalized nutrition tools and community-based digital education programs—offer promising avenues to enhance critical literacy and support informed dietary decision-making among older adults.

### 5.7 Strengths and limitations

A key strength of this study lies in its integrative application of the COM-B model, providing a comprehensive understanding of the multifactorial determinants of elderly eating behaviors, and in the novel identification of Nutritional Literacy as a cross-cutting theme. The use of in-depth, semi-structured interviews enriched the contextual understanding of participants' lived experiences.

However, limitations include a geographically confined sample, which may affect generalizability, and reliance on self-reported data, which may introduce recall or social desirability biases.

It is also important to note that cultural norms and social structures vary across regions in China. In Zhenjiang, for example, traditional values of frugality and strong family influence were particularly salient. In regions with different urbanization levels, economic development, or family dynamics, these influences may differ, potentially limiting generalizability. Future multi-site research is warranted to explore these variations.

## 6 Conclusion

This study demonstrates that elderly eating behaviors are shaped by complex, interdependent interactions among Capability, Opportunity, and Motivation domains, as conceptualized in the COM-B framework. In the digital era, Nutritional Literacy emerges as a pivotal mediator linking information environments to dietary practices.

Holistic interventions that enhance capabilities, optimize both physical and digital opportunities, and strengthen motivations are essential for promoting culturally sensitive and sustainable nutritional behaviors.

The COM-B model proved to be a robust and flexible tool for capturing these dynamics, offering insights beyond traditional behavior measurement approaches. Future interventions should continue leveraging this model to guide the development of multi-dimensional, culturally tailored nutrition strategies for older adults.

## Glossary

**Table:** 

Capability	The individual's physical and psychological capacity to engage in behavior, including knowledge, skills, and health conditions.
Opportunity	External factors that facilitate or hinder behavior, such as environmental resources, social influences, and cultural norms.
Motivation	Internal processes—both reflective (e.g., health goals) and automatic (e.g., emotional responses)—that direct and energize behavior.
COM-B Model	A theoretical framework proposing that behavior results from the dynamic interaction between Capability, Opportunity, and Motivation.
Nutritional Literacy	The ability to access, understand, evaluate, and apply nutrition-related information to make informed dietary decisions.
Restrictive Eating	The reduction or avoidance of specific foods due to health conditions, personal goals, or social influences.
Emotional Eating	Eating behavior driven by emotional states rather than physiological hunger, often in response to stress, sadness, or celebration.
External Eating	Eating behavior influenced by environmental cues, such as food availability, peer dynamics, or convenience factors.

## Statement regarding informed consent

Informed consent was obtained from all individual participants included in the study.

## Statement regarding ethical approval

All procedures performed in studies involving human participants were in accordance with the ethical standards of the Ethics Committee of the Affiliated People's Hospital of Jiangsu University (Approval No. K-2023007-Y) and with the 1964 Helsinki declaration and its later amendments or comparable ethical standards.

## CRediT authorship contribution statement

**Qian Wang:** Writing – review & editing, Writing – original draft, Investigation, Formal analysis, Data curation. **Qian Li:** Writing – review & editing, Writing – original draft, Project administration, Methodology, Investigation, Funding acquisition, Formal analysis, Data curation, Conceptualization.

## Funding

This study was supported by the Social Development Guidance Science and Technology Program of Zhenjiang, Jiangsu Province, China (Grant No.: FZ2024066).

## Declaration of competing interest

The authors declare that they have no known competing financial interests or personal relationships that could have appeared to influence the work reported in this paper.

## Data Availability

Data will be made available on request.

## Appendix A Supplementary data

Supplementary material： Overview of Themes, Subthemes, COM-B Components, and Illustrative Quotes from Semi-Structured Interviews. Image 1
