# Supplementary material for: Eating behaviors among chinese older adults: A qualitative study using the capability, opportunity, motivation, and behavior model
Source: Prev Med Rep. 2025 Jun 29;56:103156. doi: 10.1016/j.pmedr.2025.103156 (PMC12270743; doi:10.1016/j.pmedr.2025.103156)
Supplement: Supplementary file 1 — Supplementary material： Overview of Themes, Subthemes, COM-B Components, and Illustrative Quotes from Semi-Structured Interviews. [file mmc1.docx]

**Supplementary Table 1. Overview of Themes, Subthemes, COM-B Components, and Illustrative Quotes from Semi-Structured Interviews**

| **Theme** | **Subtheme** | **COM-B Component** | **Illustrative Quotes** |
| --- | --- | --- | --- |
| Restrictive Eating | Health-driven dietary adjustments (e.g., diabetes, hypertension) | Capability | "Since being diagnosed with diabetes, I have to plan my meals carefully every day." (P1, Male, 65) |
| Restrictive Eating | Chewing and digestion difficulties affecting food selection | Capability | "Because of my teeth, I can't eat anything too hard anymore, even if I want to." (P7, Female, 71) |
| Restrictive Eating | Family-imposed dietary restrictions | Opportunity | "My son controls the salt and oil at home; he says it's healthier for me." (P2, Female, 70) |
| Restrictive Eating | Cultural/religious dietary practices | Opportunity | "We always avoid meat during certain festivals, it's part of our tradition." (P12, Female, 67) |
| Restrictive Eating | Self-motivation for health preservation | Motivation | "I want to live longer for my grandchildren, so I try to eat less fried food." (P6, Male, 74) |
| Emotional Eating | Overeating in response to stress or anxiety | Capability | "When I'm upset, I find myself eating whatever snacks are around." (P5, Female, 73) |
| Emotional Eating | Nostalgic eating for emotional comfort | Motivation | "Making dumplings reminds me of happier times with my family." (P10, Female, 70) |
| Emotional Eating | Food as a celebration or reward | Motivation | "After a hard day, I reward myself with my favorite cake." (P19, Male, 65) |
| Emotional Eating | Influence of social gatherings on emotional eating | Opportunity | "During family gatherings, I always end up eating more than I should." (P11, Male, 69) |
| Nutritional Literacy | Difficulty understanding health information | Capability | "All these talks about calories and cholesterol are too complicated for me." (P5, Female, 72) |
| Nutritional Literacy | Skepticism toward online information | Capability | "There are too many health advertisements. I don't believe half of them." (P9, Female, 67) |
| Nutritional Literacy | Reliance on family or healthcare providers for information validation | Opportunity | "I always ask my daughter before trying anything new I read online." (P20, Male, 69) |
| Nutritional Literacy | Digital literacy barriers to accessing food delivery | Opportunity | "I see all those apps, but I don't know how to order. I still have to go to the market myself." (P16, Male, 72) |
| Nutritional Literacy | Anxiety and avoidance due to misinformation | Motivation | "I'm so afraid of eating the wrong thing that I sometimes just eat very little." (P14, Female, 68) |
| External Eating | Convenience-based reliance on processed foods | Capability | "Cooking for just one person is too much trouble, so I buy frozen meals." (P8, Female, 75) |
| External Eating | Lack of access to fresh produce | Opportunity | "There aren't many fresh vegetables at the market near my home." (P9, Male, 70) |
| External Eating | Financial constraints limiting healthy choices | Opportunity | "Organic food is so expensive. I usually just buy whatever is cheaper." (P13, Female, 66) |
| External Eating | Influence of dining culture and peer pressure | Motivation | "When everyone else orders rich dishes, I don't want to be the odd one out." (P4, Male, 68) |
